# Relationship between Cephalometric and Ultrasonic Airway Parameters in Adults with High Risk of Obstructive Sleep Apnea

**DOI:** 10.3390/jcm13123540

**Published:** 2024-06-17

**Authors:** Anutta Terawatpothong, Chidchanok Sessirisombat, Wish Banhiran, Hitoshi Hotokezaka, Noriaki Yoshida, Irin Sirisoontorn

**Affiliations:** 1 Department of Clinical Dentistry, Walailak University International College of Dentistry (WUICD), 87 Ranong 2 Road, Dusit, Bangkok 10300, Thailand; 2 Department of Otorhinolaryngology, Faculty of Medicine Siriraj Hospital, Mahidol University, 2 Wanglang Road, Bangkoknoi, Bangkok 10700, Thailand; 3 Department of Orthodontics and Dentofacial Orthopedics, Nagasaki University Graduate School of Biomedical Sciences, 1-7-1 Sakamoto, Nagasaki 852-8588, Japan

**Keywords:** obstructive sleep apnea, lateral cephalometry, ultrasonography

## Abstract

**Background/Objectives:** Polysomnography and cephalometry have been used for studying obstructive sleep apnea (OSA) etiology. The association between craniofacial skeleton and OSA severity remains controversial. To study OSA’s etiology, cephalometry, fiberoptic pharyngoscopy, polysomnography, and sleep endoscopy have been used; however, airway obstructions cannot be located. Recent research suggested ultrasonography for OSA screening and upper airway obstruction localization. Thus, this study aims to investigate the relationship between specific craniofacial cephalometric and ultrasonic airway parameters in adults at high risk of OSA. **Methods:** To assess craniofacial structure, lateral cephalograms were taken from thirty-three adults over 18 with a STOP-Bang questionnaire score of three or higher and a waist-to-height ratio (WHtR) of 0.5 or higher. Airway parameters were assessed through submental ultrasound. **Results:** NSBA correlated with tongue base airspace width, while MP-H correlated with oropharynx, tongue base, and epiglottis airspace width. SNA, SNB, and NSBA correlated with tongue width at the oropharynx. At tongue base, ANB and MP-H correlated with tongue width. SNB and NSBA were associated with deep tissue thickness at the oropharynx, while MP-H correlated with superficial tissue thickness at velum and oropharynx. **Conclusions:** Cephalometric parameters (SNA, SNB, ANB, NSBA, and MP-H) were correlated with ultrasonic parameters in the velum, oropharynx, tongue base, and epiglottis.

## 1. Introduction

Obstructive sleep apnea (OSA) is a breathing disorder caused by an upper airway constriction that prevents normal ventilation during sleep [1]. Respiratory effort increases to sustain airflow via a constricted airway, and this is followed by an increase in serum carbon dioxide (hypercarbia) and a drop in serum oxygen (hypoxemia). Cortical arousal is attributed to the increased work of breathing during sleep. Common symptoms of OSA include snoring, gasping, or choking respiration; frequent nighttime awakenings; excessive daytime sleepiness; and irritability. Many previous studies have reported that there are many factors that increase the risk of OSA, which include male sex, obesity (higher BMI), older age, greater neck and waist circumference, as well as craniofacial disposition and pharyngeal soft tissues [2,3,4]. Untreated obstructive sleep apnea (OSA) can lead to severe problems such as coronary artery disease, congestive heart failure, myocardial infarction, hypertension, stroke, cardiac arrhythmia, and mortality [5].

For diagnosing obstructive sleep apnea, polysomnography (PSG) is the most reliable technique [1,5]. The American Academy of Sleep Medicine (AASM) has proposed a classification system for the severity of OSA based on the apnea–hypopnea index (AHI). OSA is classified as mild to moderate (AHI ≥ 5 events/h and AHI < 30 events/h) or severe (AHI ≥ 30 events/h). Without conducting home sleep apnea testing or PSG, the use of clinical devices, questionnaires, and prediction algorithms is not recommended to diagnose OSA in adults. Uncomplicated adult patients who present with signs and symptoms indicating an increased risk of moderate-to-severe OSA can be diagnosed using home sleep apnea testing with a suitable device or PSG, while adults with comorbid conditions are recommended to use PSG for OSA diagnosis rather than home sleep apnea testing [1]. Following diagnosis, a treatment plan should be developed, which may involve behavioral therapy, oral appliances, positive airway pressure devices, surgery, and/or additional treatments. OSA is regarded as a chronic condition that needs multidisciplinary therapy [1,6].

However, polysomnography (PSG) is costly, time-consuming, many sleep laboratories have long waiting lists of patients, and an unfamiliar environment may lead to discomfort and perhaps compromise sleep quality [7]. A previous study reported that PSG, fiberoptic pharyngoscopy, and sleep endoscopy have all been used to investigate the predisposing etiological variables of OSA. Nevertheless, even with the use of all available methods, there are limitations in accurately locating the site of the blocked airway because it is not possible to mimic the sleep state in awake individuals [8]. PSG gives a range of physiological data during sleep without determining the anatomic cause of OSA. Consequently, a significant number of people remain undiagnosed due to an inability to access treatment, being asymptomatic, or lacking awareness of their condition. It is essential to place importance on the screening procedures that help in the early identification of patients at risk or those with unrecognized OSA. The screening methods for OSA involve physical examination, upper airway examination, questionnaires, and imaging technologies.

The STOP-Bang questionnaire, Berlin Questionnaire (BQ), and Epworth Sleepiness Scale (ESS) are widely used screening tools facilitating the early identification of patients at risk [9]. The STOP-Bang questionnaire is a reliable tool for screening obstructive sleep apnea in adults. It consists of four subjective items (STOP: Snoring, Tiredness, Observed Apnea, and High Blood Pressure) and four demographic criteria (Bang: BMI > 35 kg/m^2^, age > 50 years, neck circumference > 40 cm, and gender). With a high sensitivity, the STOP-Bang questionnaire can identify patients with moderate-to-severe OSA [10,11]. However, some adjustments are required for Thai patients. The Thai STOP-Bang questionnaire’s optimal BMI cutoff point was >30 kg/m^2^, which increased sensitivity at the AHI cutoff points of 5 and 15 by 88.7% and 93.2%, respectively. A patient is considered high-risk if at least three “yes” answers are provided [12]. Banhiran et al. reported that male gender and a waist-to-height ratio (WHtR) of 0.55 were significant predictors of moderate-to-severe OSA. The WHtR identifies central obesity, or visceral fat, and represents fat distribution [13]. However, some authors reported that if the WHtR is higher than 0.5 or 0.6, the risk of causing obstructive sleep apnea increases [14,15,16,17]. Therefore, the waist-to-height ratio (WHtR) should be incorporated into questionnaires for high-risk individuals to reduce the possibility of false-positive results.

Imaging technologies, such as cone-beam computed tomography (CBCT), lateral cephalometric radiographs, and ultrasonography, aid in the study of OSA. Cephalometric radiographs are routinely taken for orthodontic diagnosis and treatment planning by analyzing the relationship between the skull, maxilla, mandible, teeth, and soft tissues. The radiation dose from a lateral cephalogram is low, at approximately 5.6 μSv, whereas the CBCT is 15–26 times higher [18]. Previous research has found that most common variables, such as the angle between the anterior cranial base and the maxilla (SNA), the angle between the anterior cranial base and the mandible (SNB), the posterior airway space (PAS), the length of soft palate (PNS-P), and the distance from the hyoid bone to the mandibular plane (MP-H), may be correlated with the development and severity of OSA [19]. In addition, Banhiran et al. reported that the risk of moderate-to-severe OSA in Thai patients was also influenced by the cranial base angle (NSBA), which was 130 degrees or less. Abnormality of the skull’s base may potentially contribute to the constriction of the upper airway [20]. However, the association between the craniofacial skeleton and OSA severity remains controversial due to ethnic disparities and differences in research methodology [4,20,21,22,23].

Ultrasound has been increasingly utilized in recent years for OSA screening and upper airway obstruction localization. Ultrasonic imaging of airways has the advantages of being radiation-free, rapid, reproducible, accessible, affordable, and it can detect both anatomical and dynamic changes in pharyngeal airspace [24,25]. According to a systematic review and meta-analysis, the distance between lingual arteries (DLAs), tongue base thickness, retropalatal (RP) diameter, % RP diameter shortening during the Muller maneuver, lateral pharyngeal thickness (LPW), and upper airway (UA) length have a moderate correlation with moderate-to-severe OSA (r values ranging from 0.37 to 0.624) [26]. A prior investigation employed submental ultrasound to assess pharyngeal airway diameter alterations. Upper airway width changes in all three regions (retropalatal, oropharyngeal, and retroglossal) are correlated with the apnea–hypopnea index (AHI) and the Oxygen Desaturation Index (ODI) [27]. Furthermore, backscattered imaging analysis of standardized tongue ultrasonography reveals a significant correlation with the severity of OSA [25].

An ultrasonic device (AmCAD Biomed Corporation, Taipei City, Taiwan) calculates the risk of moderate-to-severe OSA using artificial intelligence (AI) algorithms and a clinically proven prediction method that compares the upper airway anatomical changes during normal breathing and the Muller maneuver (MM). The algorithm is constructed based on research findings that found significant anatomical features in ultrasonic parameters with the severity of obstructive sleep apnea, which results in a significant AUC and adequate sensitivity and specificity [24,27,28,29]. Utilizing the prediction model that incorporated variations in RP diameters at MM and neck circumference, the model development group achieved a sensitivity of 100% and a specificity of 65% for the independent predictors of severe OSA (AHI ≥ 30/h). The area under the curve (AUC) was 0.893 [24]. A logistic regression model was constructed utilizing the significant anatomical features and the BMI to predict the probability of moderate-to-severe OSA (AHI ≥ 15/h). The model achieved a sensitivity of 74.4%, specificity of 91.7%, and AUROC value of 0.901 [27]. The AUROC values for BMI and median dynamic change of pharyngeal diameters in detecting no-to-mild (AHI < 5/h), moderate (AHI ≥ 15/h), and severe (AHI ≥ 30/h) OSA were 0.893, 0.849, and 0.667, respectively [28]. The predictive model, utilizing multivariate analysis, produced an AUROC of 0.839 in estimating the probability of moderate-to-severe OSA (AHI ≥ 15/h). This model is based on significant features such as the narrowing of airways at the velum, oropharynx, and tongue base, as well as a substantially broader tongue and thicker deep tissue [29].

The primary objective of this study was to investigate the relationship between specific craniofacial cephalometric and ultrasonic airway parameters in adults at high risk of OSA, which are different points from those in previous reports.

## 2. Materials and Methods

### 2.1. Ethics Approval

This descriptive study was approved by the Ethics Committee at Walailak University (WUEC 24-009-01). 

### 2.2. Sample Size Calculation 

The G* Power 3.1 software (G*Power, Heinrich-Heine-Universität Düsseldorf, Düsseldorf, Germany) revealed that a minimum sample size of n = 29 is required to guarantee a sufficient statistical power of detection = 0.8 with α = 0.05 and an effect size = 0.5 (bivariate normal model). Assuming a 10% drop-out rate, the sample size will be 33.

### 2.3. Participant Selection

**Figure 1:**
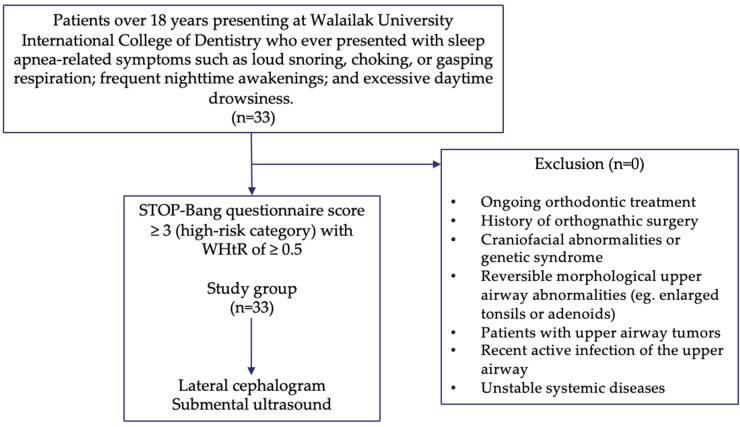
Study flow diagram. Subjects were considered high-risk if STOP-Bang scores were ≥3 according to the version used in the Department of Otorhinolaryngology, Faculty of Medicine, Siriraj Hospital, Mahidol University, Bangkok, Thailand [12].

This study was performed at a dental hospital at Walailak University International College of Dentistry. Thai individuals over 18 years old who came to the dental hospital for a regular check-up were invited to participate in this study if they had sleep apnea-related symptoms, such as loud snoring, choking, or gasping respiration; frequent nighttime awakenings; and excessive daytime drowsiness. Subjects were instructed to fill out the STOP-Bang questionnaire and assess their waist-to-height ratio (WHtR). A lateral cephalogram and submental ultrasonography was performed on patients who had a score of three or higher on the STOP-Bang questionnaire (high-risk category) [12] and WHtR of 0.5 or higher (Figure 1).

The exclusion criteria were ongoing orthodontic treatment, history of orthognathic surgery, craniofacial abnormalities or genetic syndrome, reversible morphological upper airway abnormalities such as enlarged tonsils or adenoids, upper airway tumors, recent active infection of the upper airway, and unstable systemic diseases. In this study, we recruited 33 patients (19 males, 14 females; mean age 50.06 ± 12.70 years old).

### 2.4. Data Collection

#### 2.4.1. STOP-Bang Questionnaire

**Figure 2:**
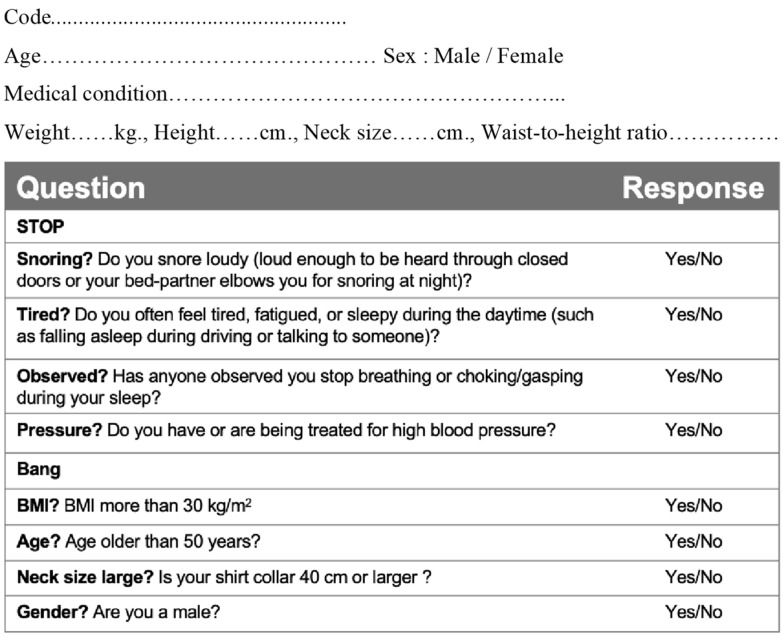
STOP-Bang questionnaire, BMI cutoff point was >30 kg/m^2^ adjusted for Thais [12].

Participants’ demographics (sex, age, weight, height, BMI, waist size, WHtR, and neck size) were gathered. The questionnaire asks yes-or-no questions to assess OSA risk (Figure 2). A patient is regarded as being at low risk for OSA if there are no more than two yes answers on the questionnaire, and high risk if there are three or more yes answers [12]. A total of 33 participants who had a score of three or higher, with a waist-to-height ratio (WHtR) of 0.5 or higher, had data on their craniofacial structure gathered via lateral cephalograms and their airway parameters were evaluated using submental ultrasounds.

#### 2.4.2. Lateral Cephalometric Radiograph

Lateral cephalometric radiographs were taken using the Orthophos XG5DS machine from Sirona Dental Systems in Bensheim, Germany. At values of 63–69 kV and 8–12 mA, a cephalometric unit was implemented. Cephalometry was conducted in an upright position, with the head held to the cephalostat using ear rods and supported by the forehead to verify the natural head position. All cephalometric radiographs used the same 1.1 magnification factor. The teeth were in maximum intercuspation, the lips were at rest, and the head was in a natural head posture. The lateral cephalometric radiographs were reproduced on paper with a 1:1 scale in millimeters.

#### 2.4.3. Anatomical Landmarks and Measurement

**Figure 3:**
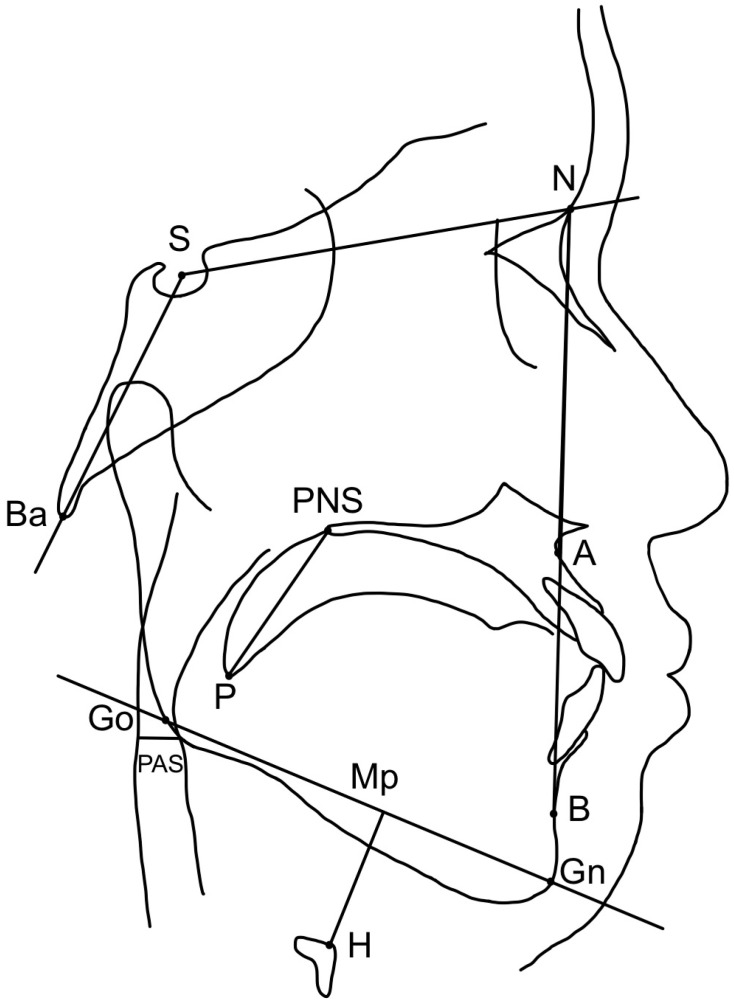
Cephalometric landmarks and parameters. Landmarks: S, sella; N, nasion; Ba, basion; A, subspinale; B, supramental; Go, Gonion; Gn, Gnathion; MP, mandibular plane; P, tip of soft palate; H, hyoid; PNS, posterior nasal spine; PAS, posterior airway space. Parameters: SNA (degree), antero-posterior position of the maxilla relative to the anterior cranial base; SNB (degree), antero-posterior position of the mandible relative to the anterior cranial base; ANB (degree), the difference between SNA and SNB; NSBA (degree), angle formed by nasion–sella–basion; MP-H (mm), perpendicular distance from hyoid bone to mandibular plane (vertical position of hyoid bone); PAS (mm), retroglossal posterior airway space, defined as the shortest distance between base of tongue and posterior pharyngeal wall; PNS-P (mm), soft palate length, measured from the posterior nasal spine (PNS) to the tip of soft palate (P).

Figure 3 displays the landmarks and measurement nomenclatures. The intra-class correlation coefficient was used to measure intra-examiner reliability. All parameters were evaluated twice, two weeks apart, by the same investigator.

#### 2.4.4. Submental Ultrasound

The FDA-approved Terason uSmart3200T (K193510; Teratech Corporation, Burlington, MA, USA) ultrasound scanner with a convex transducer (5C2A) was used to acquire radiofrequency ultrasound signals and B-mode images. AmCAD-UO (K162574, AmCad BioMed Corporation, Taipei City, Taiwan) has been used for analyzing radiofrequency ultrasound signals. This machine calculates the probability of moderate-to-severe OSA using artificial intelligence (AI) algorithms and a clinically proven prediction method that compares the upper airway anatomical changes during normal breathing and the Muller maneuver (MM). The algorithm is constructed based on research findings that found significant anatomical features in ultrasonic parameters with the severity of obstructive sleep apnea (OSA), which results in a significant AUC and adequate sensitivity and specificity. 

**Figure 4:**
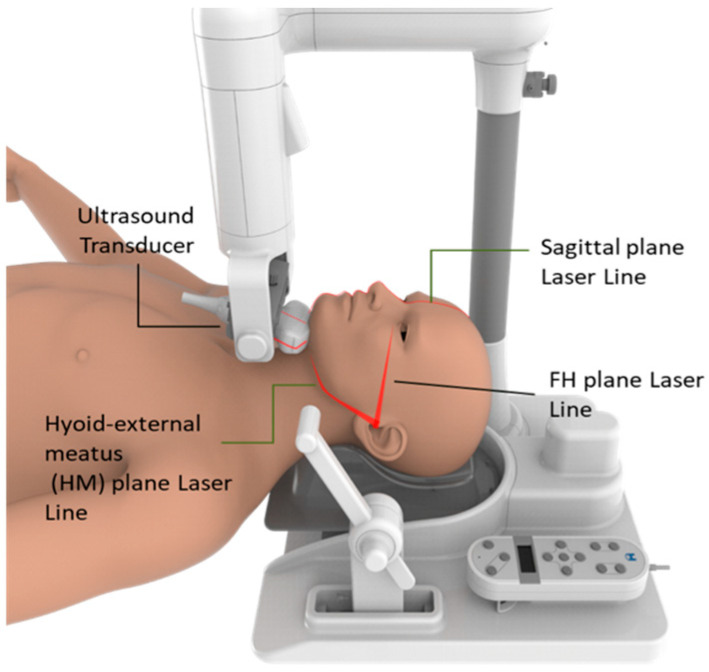
Submental ultrasonography equipment, laser alignment (AmCad BioMed Corporation).

Prior to scanning, laser beams (FDA Establishment Registration & Device Listing No. 3015218501; AmCad BioMed Corporation, Taipei City, Taiwan) were used for reproducible reasons and to set up an individual in the supine position, along with the sagittal plane, Frankfort horizontal plane (FH plane), and a cross-sectional plane through the hyoid bone and the external acoustic meatus (HM plane) (Figure 4). 

**Figure 5:**
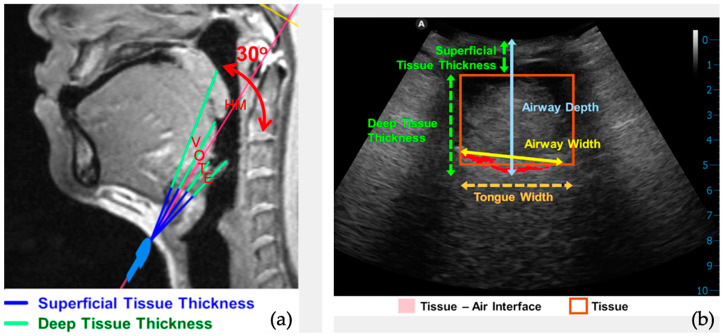
(**a**) The 30° segment of the upper airway; HM, hyoid-external meatus. (**b**) Transverse view of ultrasonographic images.

The ultrasonic transducer is aligned with the HM plane’s laser projection in the submental region to provide transverse, cross-sectional images (Figure 4). Three replicates were automatically scanned by an ultrasonic transducer during tidal breathing and Muller maneuvers at 30 degrees, covering the velum (V), the oropharynx (O), the base of the tongue (T), and the epiglottis (E) (Figure 5a). 

During scanning, patients were instructed to alternate between regular breathing and Muller maneuvers, completing a total of three repetitions. Muller maneuvers were conducted by inhaling vigorously with the mouth and nose close, simulating upper airway obstruction during awake. 

The airway’s width, airway’s depth, tongue width, airspace–tissue width ratio, superficial tissue thickness, and deep tissue thickness were measured (Figure 5b). Dynamic changes in airway width during tidal breathing and Muller maneuvers, or percentages of airway contraction, were calculated as a percentage of pharyngeal diameters shortening from the diameters during tidal breathing to that during MM. 

The risk of moderate-to-severe OSA (AHI ≥ 15/h) was evaluated as a percentage based on the assessment of ultrasonic parameters. The risk of 0–40% is classified as low, 40–80% is classified as medium, and 80–100% is classified as a high risk of moderate-to-severe OSA [24,27,28,29].

### 2.5. Data Analysis Strategies

The data were processed with SPSS version 25 software (IBM SPSS, Armonk, NY, USA), and the intra-examiner reliability was assessed using the intra-class correlation coefficient (ICC) for absolute agreement. Descriptive statistics were utilized to present the average values and standard deviations (S.D.). To establish data normality, the Shapiro–Wilk test was utilized. The correlation between cephalometric parameters and ultrasonic airway parameters was calculated using Spearman’s rho. Statistical significance was assessed at *p* < 0.05 in a two-tailed test.

## 3. Results

There were 33 adults; men were 57.60% (n = 19) and women were 42.40% (n = 14) of the patients. The mean age was 50.06 ± 12.70 years, weight was 77.61 ± 13.82 kg, height was 166.30 ± 8.60 cm, BMI was 28.00 ± 4.40 kg/m^2^, waist was 93.12 ± 9.19 cm, waist-to-height ratio (WHtR) was 0.56 ± 0.05, neck size was 38.89 ± 3.59 cm, and STOP-Bang score was 4.30 ± 1.10. These results are summarized in Table 1. The mean cephalometric parameters and ultrasound parameters are shown in Table 2 and Table 3, respectively. The repeated radiographic measurements exhibited excellent reliability, as determined by intra-class correlation coefficients (ICCs) that varied between 0.978 and 0.998.

Regarding the correlation between cephalometric measurements and ultrasound measurements for assessing the risk of moderate-to-severe obstructive sleep apnea (OSA), no statistically significant relationship was found between them (Table 4).

**Figure 6:**
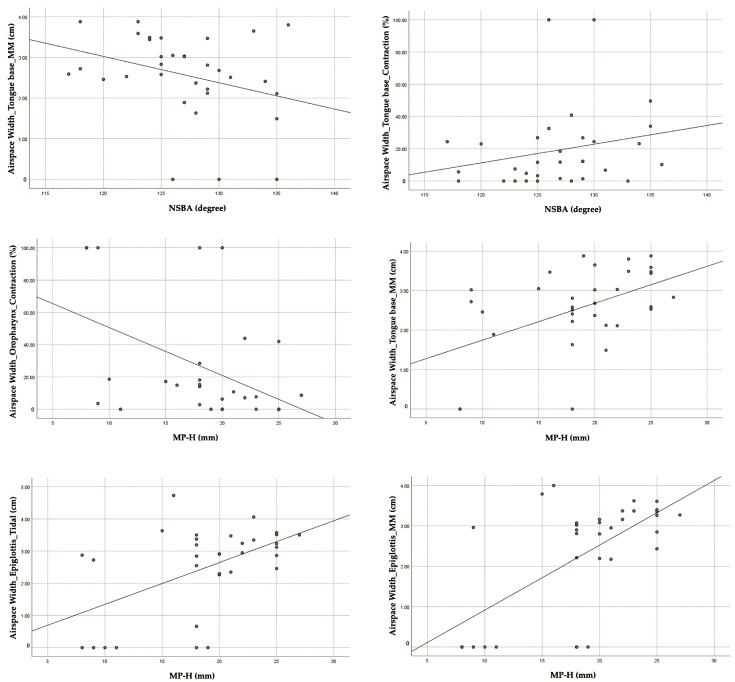
The scatter plots of significant correlation between cephalometric parameters and ultrasound parameters (airspace width).

The correlation between ultrasound parameters (airspace width) and cephalometric parameters, NSBA, was determined to be significant at the tongue base region during Muller maneuvers (r = −0.377, *p* < 0.05) and contraction (r = 0.354, *p* < 0.05). Furthermore, MP-H exhibited significant correlations with airspace width at the oropharynx during contraction (r = −0.471, *p*-value < 0.01), tongue base during Muller maneuvers (r = 0.439, *p*-value < 0.05), epiglottis during tidal breathing (r = 0.423, *p*-value < 0.05), and epiglottis during Muller maneuvers (r = 0.518, *p*-value < 0.01) (Table 4). The correlation scatter diagrams are illustrated in Figure 6.

**Figure 7:**
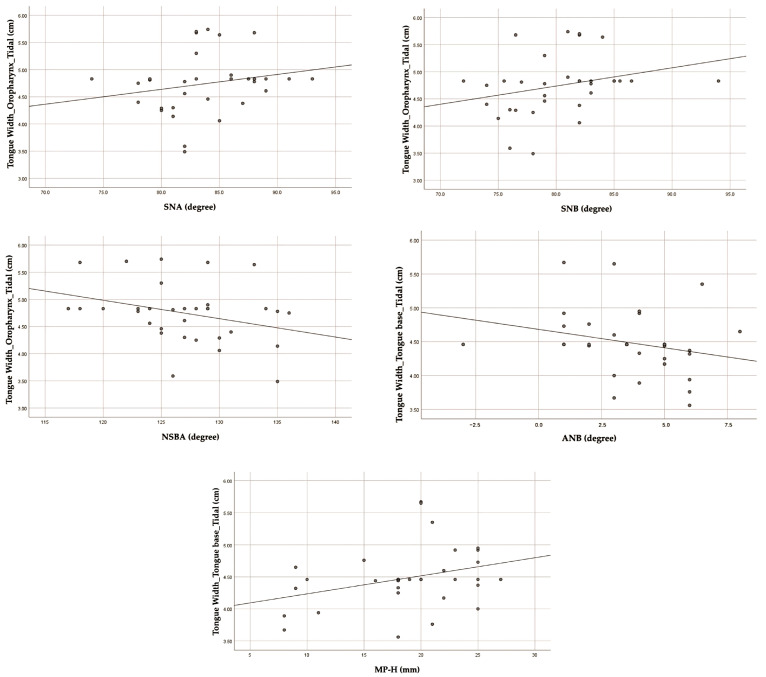
The scatter plots of significant correlation between cephalometric parameters and ultrasound parameters (tongue width).

According to the correlation between cephalometric parameters and ultrasound parameters (tongue width), there were significant relationships between the following cephalometric parameters: SNA, SNB, and NSBA, and tongue width at the oropharynx (r = 0.357, *p*-value < 0.05; r = 0.384, *p*-value < 0.05; r = −0.358, *p*-value < 0.05, respectively). Additionally, ANB and MP-H correlated significantly with tongue width at the tongue base (r = −0.355, *p*-value < 0.05; r = 0.355, *p*-value < 0.05, respectively), as reported in Table 4. The scatter plots of the correlation are shown in Figure 7.

There was no statistically significant relationship between cephalometric measurements and airspace–tissue width ratio, as reported in Table 4.

**Figure 8:**
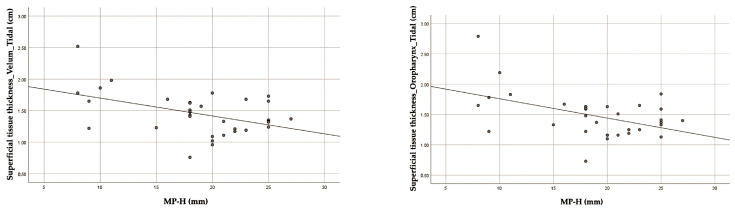
The scatter plots of significant correlation between cephalometric parameters and ultrasound parameters (superficial tissue thickness).

This study found a significant association between the cephalometric parameter MP-H and superficial tissue thickness at the velum and oropharynx during tidal breathing. The correlation coefficients were −0.348 and −0.353, with *p*-values < 0.05, respectively. These results are presented in Table 4. The correlation scatter plots are shown in Figure 8.

**Figure 9:**
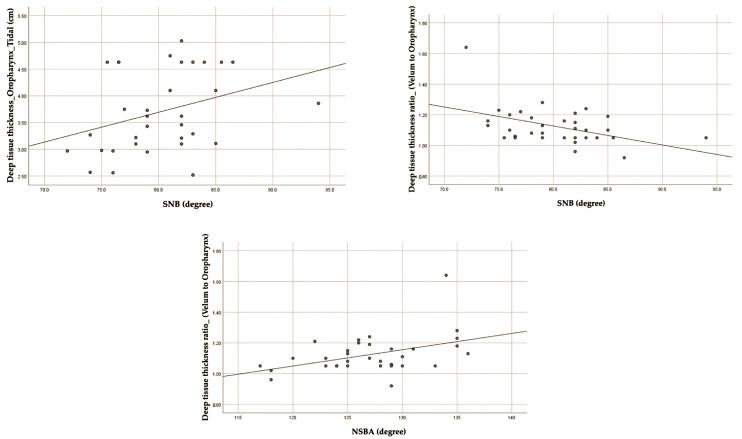
The scatter plots of significant correlation between cephalometric parameters and ultrasound parameters (deep tissue thickness).

The correlation between cephalometric parameters and deep tissue thickness is reported in Table 4. SNB was found to be significantly correlated with deep tissue thickness during tidal breathing and deep tissue thickness ratio at the oropharynx (r = 0.367, *p*-values < 0.05 and r = −0.384, *p*-values < 0.05, respectively). Moreover, this study revealed a significant correlation between NSBA and deep tissue thickness ratio at the oropharynx (r = 0.389, *p* < 0.05). Figure 9 illustrates the scatter plots of the correlation.

Therefore, the main finding of this study was the correlation of the cephalometric parameters NSBA and airspace width at the tongue base, while MP-H correlated with airspace width at the oropharynx, tongue base, and epiglottis. Regarding tongue width, there was a correlation with SNA, SNB, and NSBA at the oropharynx, while ANB and MP-H showed a correlation at the tongue base. For superficial tissue thickness, MP-H showed a correlation at the velum and oropharynx. Moreover, according to deep tissue thickness, SNB and NSBA found a correlation at the oropharynx.

## 4. Discussion

Polysomnography, fiberoptic pharyngoscopy, sleep endoscopy, and cephalometry have all been used to investigate the predisposing etiological variables of OSA. However, despite utilizing all available methods, accurately locating the site of the blocked airway is limited because it is not possible to mimic the sleep state in awake individuals [8]. Additionally, PSG is time-consuming, uncomfortable, which may compromise sleep quality, and costly [31]. Therefore, some patients remain undiagnosed. On the contrary, the ultrasound imaging of airways has the advantages of being radiation-free, rapid, reproducible, accessible, affordable, and it can identify both structural and functional changes in the pharyngeal airways [24,25,26,27,32]. Therefore, it can enhance the early detection of those with unrecognized OSA.

In terms of imaging technology that helps in studying OSA, a cephalometric radiograph is routinely taken for orthodontic diagnosis. Although several studies had reported the association between the craniofacial skeleton and OSA severity, the results remain controversial due to ethnic disparities and differences in research methodology [4,19,20,21,22,23]. In addition, no previous research has investigated the relationship between craniofacial cephalometric and ultrasonic airway parameters in adults. Accordingly, the objective of this study was to investigate the relationship between specific craniofacial cephalometric and ultrasonic airway parameters in adults at high risk of OSA.

Regarding the correlation between cephalometric measurements and ultrasound measurements for assessing the risk of moderate-to-severe obstructive sleep apnea (OSA), no statistically significant relationship was found between them. This finding contradicts prior research that proposed a significant association between specific cephalometric parameters and the severity of OSA [4,19,20,21,22,23]. This might be due to varying methods for measurement; the risk percentage of moderate-to-severe OSA (AHI ≥ 15/h) in our study was based on ultrasonic parameters using artificial intelligence (AI) algorithms. However, others [4,19,20,21,22,23] reported the correlation of cephalometric data with OSA severity based on the apnea–hypopnea index (AHI).

In relation to the correlation between ultrasound parameters (airspace width) and cephalometric parameters, NSBA was determined to be significant at the tongue base region. Moreover, MP-H exhibited significant correlations with airspace width at the oropharynx, tongue base, and epiglottis. Our research found obstruction sites in the oropharynx, tongue base, and epiglottis, which correlated with airspace width, while a recent study reported that patients with moderate-to-severe OSA (AHI ≥ 15) had reduced airspace in the velum, oropharynx, and tongue base region [29]. Our study presents a novel finding as it uses airspace width as the measurement variable to correlate with cephalometric parameters. However, cephalometric data [4,19,20,21,22,23] and ultrasonic parameters [29] have been reported in prior research as being correlated with the apnea–hypopnea index (AHI). This study did not conduct polysomnography; therefore, the relationship’s direction (positive or negative) (Figure 6) does not correspond with the severity of OSA. Increased airspace width might not represent a lower risk of moderate-to-severe OSA since other factors such as superficial and deep tissue thickness, tongue volume, tongue position, and skeletal structure all influence the development of OSA. A previous study on MRI found that the thickness of the lateral pharyngeal wall has been suggested to be the anatomical factor influencing airway constriction in OSA patients [33].

According to a novel finding of the correlation between cephalometric parameters and ultrasound parameters (tongue width), there was a positive correlation between tongue width at the oropharynx and both SNA and SNB; conversely, a negative correlation was observed with NSBA. Furthermore, an analysis revealed a positive correlation between tongue base width and MP-H but a negative correlation with ANB (Figure 7).

In our study, SNA was 83.40 ± 4.19 degrees in males and 84.57 ± 4.29 degrees in females, whereas SNB was 80.34 ± 5.03 degrees in males and 80.07 ± 3.81 degrees in females (Table 2). This finding indicates that a normal position or protruding maxilla and mandible may be associated with increased tongue width in the oropharynx region (Figure 7). This is supported by a previous study that found that the maxilla–mandibular relationship and tongue volume were significantly correlated [34]. 

The ANB value was 3.05 ± 2.33 degrees in males and 4.50 ± 1.78 degrees in females (Table 2), representing high prevalence in skeletal Class I and Class II patterns. Our findings also suggest that the skeletal Class II pattern has a smaller tongue base width than the skeletal Class I pattern (Figure 7). This is supported by previous studies, which found that skeletal Class II patients had a smaller tongue body [35,36], lower tongue posture, and a higher incidence of posterior inferior hyoid bone position [35]. Therefore, a normal maxillomandibular relationship or small tongue width may not indicate a lower risk of OSA. This might be explained by OSA, a multifactorial syndrome involving various anatomical variables and neuromuscular adaptations. The more inferior positions of the tongue, greater tongue thickness or volume, and fat tissue thickness all contributed to the chance of developing OSA. Furthermore, our findings revealed a greater skeletal discrepancy in the ANB value of female participants. In agreement with the study of adult patients with OSA in Korea, Kim S-J et al. reported that the prevalence of hyperdivergent Class II skeletal patterns was more prevalent among females, regardless of the severity of the disease [37]. However, a researcher [38] reported a lower AHI in females compared to males. This discrepancy may be attributed to the skeletal structure being influenced by sex or ethnic differences, the prevalence of obesity, and differences in anatomical structure, which lead to the development of OSA.

The NSBA for men was 126.89 ± 5.44 degrees, while for women it was 127.29 ± 4.65 degrees, which is less when compared with the normative values of Thai adults without clinical features of OSAS (Table 2). The findings of our study indicated a negative correlation between tongue width in the oropharynx and NSBA (Figure 7). As a previous study suggested, in those with Class II malocclusion, the cranial base angle (NSBA) has shown a significant increase [39]. Consequently, a smaller tongue width in the oropharynx may be associated with a greater NSBA in the skeletal Class II pattern, in agreement with prior studies [35]. Moreover, our mean value of NSBA reported the same direction as the previous study, which suggested that a smaller NSBA angle of 130 degrees or less increases the possibility of having moderate-to-severe OSA [20].

Our study found that the MP-H was 20.37 ± 4.23 mm in men and 16.64 ± 6.36 mm in women. This is higher than the normal values found in Thai adults without OSAS clinical features (Table 2). MP-H positively correlated significantly with tongue base width (Figure 7). This might be owing to a larger tongue; it may extend caudally, displacing the hyoid downward, thereby increasing the mandibular-to-hyoid distance (MP-H), which is in accordance with previous reports [40,41]. In addition, our mean value of MP-H distance reported agreement with a previous study, which proposed that 18 mm or greater of MP-H enhances the chance of having moderate-to-severe OSA. This is because the inferior position of the hyoid causes the tongue to move backwards, which contributes to the collapse of the airway [20]. 

Our research found obstruction sites in the oropharynx and tongue base correlated with tongue width. In accordance with a recent study, it was found that patients with moderate-to-severe OSA (AHI ≥ 15) had wider tongues in the velum, oropharynx, and tongue base region [29].

Additionally, a previous study reported that measuring tongue base width by determining the distance between the lingual arteries (DLAs) to be over 30 mm influences the risk of moderate-to-severe obstructive sleep apnea [42]. In our study, tongue base width was 45.7 mm on average (Table 3), which is in accordance with prior findings. 

There were no significant correlations between airspace–tissue width ratio and any of the cephalometric parameters. These indicate that the airspace–tissue width ratio may not relate to craniofacial structure in patients with a high risk of OSA. However, a recent study found that patients with moderate-to-severe OSA (AHI ≥ 15) showed a strong correlation with the airspace–tissue width ratio in the velum region [29]. This may be attributed to discrepancies in the variables used in the correlation analysis.

Our findings revealed a negative relationship between MP-H and superficial tissue thickness at the velum and oropharynx (Figure 8). The superficial tissues were mainly subcutaneous fat and suprahyoid muscles. Therefore, fat deposition might be influenced by submandibular configuration. On the contrary, previous studies have discovered that patients with moderate-to-severe OSA had thicker superficial and deep tissue in the oropharynx region. This may be attributed to lower position of the hyoid bone [29]. This might be due to ethnic heterogeneity in craniofacial structure.

At the oropharynx, SNB was negatively correlated with the deep tissue thickness ratio but positively correlated with deep tissue thickness. In addition, a study revealed a positive correlation between NSBA and the deep tissue thickness ratio at the oropharynx (Figure 9). The soft palate, tongue, or epiglottis comprised the deep tissue thickness, based on the region being imaged. The thickness of the deep tissue, primarily in the tongue, in the oropharynx region had increased in patients with moderate-to-severe OSA [29]. Therefore, this could be the increased SNB effect on tongue thickness in the oropharynx, which is supported by a prior study that discovered a substantial correlation between the maxilla–mandibular and tongue volume [34]. The ratio of deep tissue thickness, mainly the tongue, between the oropharynx and the velum was found to be related to the severity of OSA. In other words, an increase in deep tissue thickness in the oropharynx is found in patients with moderate-to-severe OSA. The ratio of deep tissue thickness defined the morphology of the tongue [29]. Consistent with our research findings, a reduction in the ratio of deep tissue thickness in the oropharynx was found to be associated with an increase in SNB, which consequently influenced the thickness of the tongue. The previous study reported a larger tongue body found in the skeletal Class I pattern [35] with a decreased NSBA angle [39]. Banhiran et al. reported that a smaller NSBA angle of 130 degrees or less increases the possibility of having moderate-to-severe OSA [20]. Consistent with our findings, we found a reduced NSBA associated with a decrease in the ratio of deep tissue thickness or an increase in deep tissue thickness (tongue thickness).

Nevertheless, our analysis revealed that there are no statistically significant disparities between PAS, PNS-P, and ultrasonic parameters. This might be attributed to variations in the methodology of studies and the variety of ethnicities affecting craniofacial structure.

## 5. Limitations

There were some potential limitations of this study. Cephalometry is conducted when the patient is awake and standing, excluding data collected during sleep when the person is laying down with relaxed pharyngeal muscles. Lateral cephalograms represent craniofacial components in two dimensions, with indistinct soft tissue measurements and no information on the transverse pharyngeal dimensions. Submental ultrasonography can only assess the transverse pharyngeal dimension, not the anteroposterior (AP) diameter, and has limits for determining nasal obstruction. However, the ultrasonography was conducted during wakefulness. During sleep, sleep stages and sleep position may affect the result. 

This is a preliminary study; we collected specific cephalometric parameters based on previous research that has variations in the methods of measurement. This study did not perform polysomnography; therefore, the result may not correspond to the severity of OSA in terms of the apnea–hypopnea index (AHI). A future study should include a larger sample size with a control group and include the heterogeneity of severity, which may be useful in subsequent comparisons across groups. Polysomnography should be performed to verify the results from ultrasonic findings. Additionally, including more cephalometric variables in both antero-posterior and vertical dimensions, such as soft tissue parameters related to the same area with submental ultrasound, is required to evaluate the influence of parameters and acquire a better understanding. Cone-beam CT (CBCT) can help clarify the airway structure in three dimensions, and other sleep tests, such as drug-induced sleep/sedation endoscopy (DISE), should be conducted to evaluate anatomical factors affecting upper airway collapsibility in the same area of the velum (V), oropharynx (O), tongue base (T), and epiglottis (E), so that the study findings are the most reliable.

This study was conducted at a dental hospital among a general population, resulting in a relatively low STOP-Bang score. It is important to note that the findings may vary if the study were to be conducted in a sleep clinic setting.

## 6. Conclusions

- This is the first study to investigate the relationship between specific cephalometric and ultrasonic airway parameters in adults at high risk of obstructive sleep apnea. We discovered that SNA, SNB, ANB, NSBA, and MP-H were significantly associated with ultrasonic airway parameters in the velum, oropharynx, tongue base, and epiglottis.
- A normal maxillomandibular relationship or small tongue width may not indicate a lower risk of OSA. Various anatomical factors, such as inferior positions of the tongue, greater tongue thickness or volume, skeletal structure, and superficial and deep tissue thickness, contributed to the chance of developing OSA.
- Combining several aspects of information, such as questionnaires, cephalometry, and submental ultrasonography, together with medical history allows us to prioritize individuals with unrecognized OSA for further sleep evaluation or therapy.

## Figures and Tables

**Table 1:** Demographic data of subjects.

Characteristic (n = 33)	Mean ± S.D.
Age (year)	50.06 ± 12.70
Weight (kg)	77.61 ± 13.82
Height (cm)	166.30 ± 8.60
Body Mass Index (BMI) (kg/m^2^)	28.00 ± 4.40
Waist (cm)	93.12 ± 9.19
Waist-to-height ratio (WHtR)	0.56 ± 0.05
Neck size (cm)	38.89 ± 3.59
STOP-Bang score	4.30 ± 1.10

The data are presented as mean ± standard deviation (S.D.).

**Table 2:** Cephalometric data of subjects and normative values of Thai adults without clinical features of OSAS [30].

Cephalometric Parameters	This Study	Thai Norms (Somboonsap N.)		
	Male	Female	Male	Female
	Mean ± S.D.	Mean ± S.D.	Mean ± S.D.	Mean ± S.D.
SNA (degree)	83.40 ± 4.19	84.57 ± 4.29	84.3 ± 4.0	84.4 ± 3.1
SNB (degree)	80.34 ± 5.03	80.07 ± 3.81	81.5 ± 4.1	80.7 ± 3.2
ANB (degree)	3.05 ± 2.33	4.50 ± 1.78	3.2 ± 1.8	3.9 ± 2.0
NSBA (degree)	126.89 ± 5.44	127.29 ± 4.65	130.8 ± 5.3	132.2 ± 6.0
MP-H (mm)	20.37 ± 4.23	16.64 ± 6.36	16.1 ± 5.3	10.8 ± 4.9
PAS (mm)	10.58 ± 3.81	9.36 ± 2.21	14.2 ± 3.4	11.1 ± 3.3
PNS-P (mm)	40.79 ± 2.86	37.07 ± 2.81	34.8 ± 6.1	32.3 ± 3.1

The data are presented as mean ± standard deviation (S.D.).

**Table 3:** Ultrasonic data of subjects.

Ultrasound Parameters	Mean ± S.D.
OSA Risk Assessment (%)	83.57 ± 16.04
Airspace Width (cm)	
Velum	
Tidal	2.07 ± 1.77
Muller	1.37 ± 1.68
Contraction	37.07 ± 42.15
Oropharynx	
Tidal	2.51 ± 1.42
Muller	2.01 ± 1.59
Contraction	26.04 ± 37.12
Tongue base	
Tidal	2.65 ± 1.34
Muller	2.14 ± 1.35
Contraction	21.37 ± 28.01
Epiglottis	
Tidal	2.17 ± 1.53
Muller	1.95 ± 1.54
Contraction	13.70 ± 30.21
Tongue Width (cm)	
Velum	5.12 ± 0.49
Oropharynx	4.82 ± 0.60
Tongue base	4.57 ± 0.58
Epiglottis	4.29 ± 0.51
Airspace–Tissue Width Ratio	
Velum	0.40 ± 0.34
Oropharynx	0.54 ± 0.32
Tongue base	0.60 ± 0.31
Epiglottis	0.52 ± 0.37
Superficial Tissue Thickness (cm)	
Velum	
Tidal	1.45 ± 0.34
Oropharynx	
Tidal	1.48 ± 0.37
Ratio (Velum to region)	0.98 ± 0.08
Tongue base	
Tidal	1.56 ± 0.25
Ratio (Velum to region)	0.93 ± 0.11
Epiglottis	
Tidal	1.60 ± 0.25
Ratio (Velum to region)	0.89 ± 0.16
Deep Tissue Thickness (cm)	
Velum	
Tidal	4.23 ± 0.68
Oropharynx	
Tidal	3.71 ± 0.76
Ratio (Velum to region)	1.12 ± 0.12
Tongue base	
Tidal	3.32 ± 0.70
Ratio (Velum to region)	1.28 ± 0.16
Epiglottis	
Tidal	3.25 ± 0.57
Ratio (Velum to region)	1.33 ± 0.18

The data are presented as mean ± standard deviation (S.D.).

**Table 4:** Correlation coefficient of cephalometric parameters and ultrasound parameters (OSA risk assessment, airspace width, tongue width, airspace–tissue width ratio, superficial tissue thickness, and deep tissue thickness).

Cephalometric Parameters/Ultrasound Parameters	SNA	SNB	ANB	NSBA	MP-H	PAS	PNS-P
**OSA Risk Assessment (%)**	−0.046	−0.093	0.127	0.136	−0.087	0.122	−0.034
**Airspace Width (cm)**							
Velum							
Tidal	0.044	0.125	−0.205	−0.173	0.108	−0.168	0.103
Muller	0.073	0.077	−0.052	−0.193	0.12	−0.128	−0.026
Contraction	−0.174	−0.179	−0.119	0.177	−0.114	0.17	0.111
Oropharynx							
Tidal	−0.134	−0.13	0.01	−0.003	−0.013	−0.047	0.301
Muller	0	0.062	−0.101	−0.204	0.273	−0.163	0.068
Contraction	−0.103	−0.158	0.011	0.24	−0.471 **	0.195	0.302
Tongue base							
Tidal	0.023	0.015	0.021	−0.172	0.306	−0.032	0.218
Muller	0.212	0.222	0.009	−0.377 *	0.439 *	−0.097	0.005
Contraction	−0.229	−0.269	0.05	0.354 *	−0.242	0.031	0.194
Epiglottis							
Tidal	−0.069	−0.178	0.164	−0.104	0.423 *	0.044	0.04
Muller	−0.136	−0.126	−0.054	−0.134	0.518 **	0.063	0.005
Contraction	0.238	0.008	0.364	0.135	−0.023	−0.154	−0.011
**Tongue Width (cm)**							
Velum	0.159	0.209	−0.173	−0.194	0.327	0.157	0.044
Oropharynx	0.357 *	0.384 *	−0.172	−0.358 *	0.272	0.032	−0.059
Tongue base	−0.011	0.144	−0.355 *	−0.043	0.355 *	0.003	0.112
Epiglottis	0.05	0.048	−0.038	0.063	0.22	0.033	0.008
**Airspace–Tissue Width Ratio**							
Velum	0.018	0.072	−0.127	−0.077	−0.01	−0.249	0.11
Oropharynx	−0.213	−0.228	0.055	0.035	−0.142	−0.076	0.3
Tongue base	−0.044	−0.117	0.186	−0.155	0.193	0.054	0.207
Epiglottis	−0.092	−0.151	0.108	−0.051	0.299	0.166	0.135
**Superficial Tissue Thickness**							
Velum							
Tidal	0.141	0.16	−0.008	−0.173	−0.348 *	0.109	0.121
Oropharynx							
Tidal	0.096	0.141	−0.065	−0.139	−0.353 *	0.262	0.235
Ratio (Velum to region)	0.244	0.134	0.287	−0.034	−0.012	−0.282	−0.13
Tongue base							
Tidal	0.155	0.213	−0.094	−0.166	−0.303	0.193	0.267
Ratio (Velum to region)	0.019	−0.035	0.152	−0.021	−0.238	−0.028	0.1
Epiglottis							
Tidal	0.19	0.222	−0.001	−0.197	−0.239	0.117	0.142
Ratio (Velum to region)	0.014	0.006	0.003	−0.018	−0.194	0.018	0.212
**Deep Tissue Thickness**							
Velum							
Tidal	0.27	0.307	−0.193	−0.088	0.061	−0.225	−0.002
Oropharynx							
Tidal	0.307	0.367 *	−0.17	−0.139	0.12	−0.024	−0.108
Ratio (Velum to region)	−0.327	−0.384 *	0.176	0.389 *	−0.271	0.014	0.021
Tongue base							
Tidal	0.129	0.177	−0.18	0.03	−0.016	−0.276	−0.116
Ratio (Velum to region)	−0.044	−0.136	0.225	−0.014	0.177	0.218	0.01
Epiglottis							
Tidal	0.242	0.226	−0.052	0.072	−0.015	−0.083	0.003
Ratio (Velum to region)	0.074	0.091	−0.049	−0.202	0.217	0.118	0.127

* Correlation is significant at the 0.05 level, ** Correlation is significant at the 0.01 level (2-tailed).

## Data Availability

Data is contained within the article.
